# Death of a Medical Editor

**Published:** 1911-09-30

**Authors:** 


### Death of a Medical Editor.

The death is announced of Dr. W. Langford Symes, formerly a well-known specialist in the diseases of children at Dublin. He was for many years the Editor of the *Journal of the Irish Medical Association.* During the past few years he had been in practice at Llandrindod Wells, to which place he had gone on account of his health. Dr. Symes qualified in Dublin in 1883 and at first practiced in county Wicklow, but coming to London he took up the study of children's diseases at Great Ormond Street, and then returned to Dublin, where he obtained the Fellowship of the Royal College of Physicians in 1898. In 1902 he took the M.D. of the University of Durham.

